# Unique Regulatory Properties of Mesangial Cells Are Genetically Determined in the Rat

**DOI:** 10.1371/journal.pone.0111452

**Published:** 2014-10-24

**Authors:** Ping-Chin Lai, Ling-Yin Chiu, Prashant Srivastava, Cristina Trento, Francesco Dazzi, Enrico Petretto, H. Terence Cook, Jacques Behmoaras

**Affiliations:** 1 Kidney Institute, Department of Nephrology, School of Medicine, Chang Gung University, Chang Gung Memorial Hospital, Taipei, Taiwan; 2 Centre for Complement and Inflammation Research (CCIR), Hammersmith Hospital, Imperial College London, London, United Kingdom; 3 MRC Clinical Sciences Centre, Faculty of Medicine, Imperial College London, London, United Kingdom; 4 Stem Cells Biology, Department of Medicine, Imperial College London, London, United Kingdom; Universidade de Sao Paulo, Brazil

## Abstract

Mesangial cells are glomerular cells of stromal origin. During immune complex mediated crescentic glomerulonephritis (Crgn), infiltrating and proliferating pro-inflammatory macrophages lead to crescent formation. Here we have hypothesised that mesangial cells, given their mesenchymal stromal origin, show similar immunomodulatory properties as mesenchymal stem cells (MSCs), by regulating macrophage function associated with glomerular crescent formation. We show that rat mesangial cells suppress conA-stimulated splenocyte proliferation *in vitro*, as previously shown for MSCs. We then investigated mesangial cell-macrophage interaction by using mesangial cells isolated from nephrotoxic nephritis (NTN)-susceptible Wistar Kyoto (WKY) and NTN-resistant Lewis (LEW) rats. We first determined the mesangial cell transcriptome in WKY and LEW rats and showed that this is under marked genetic control. Supernatant transfer results show that WKY mesangial cells shift bone marrow derived macrophage (BMDM) phenotype to M1 or M2 according to the genetic background (WKY or LEW) of the BMDMs. Interestingly, these effects were different when compared to those of MSCs suggesting that mesangial cells can have unique immunomodulatory effects in the kidney. These results demonstrate the importance of the genetic background in the immunosuppressive effects of cells of stromal origin and specifically of mesangial cell-macrophage interactions in the pathophysiology of crescentic glomerulonephritis.

## Introduction

Macrophage infiltration plays a crucial role in the development of crescentic glomerulonephritis [1]–[3]. Under pathologic conditions, activated macrophages secrete a variety of inflammatory mediators and stimulate resident cells such as mesangial cells (MCs) toward activation and survival [4], [5]. Once activated, MCs express chemokines (i.e monocyte chemoattractant protein 1; MCP-1) and accelerate accumulation of macrophages, leading to progression of glomerular injury [6], [7].

Depletion of macrophages [8] or inhibition of mesangial cell proliferation and activation [9], [10] attenuates glomerular injury in rodent models of glomerulonephritis, suggesting that both macrophage and mesangial cell function contribute to the progression of renal injury in glomerulonephritis. We have been investigating the relative role of MCs and macrophages in the nephrotoxic nephritis (NTN) model of crescentic glomerulonephritis (Crgn) in the Wistar Kyoto (WKY) rat. This inbred rat strain is uniquely susceptible to macrophage-dependant NTN [11]. Bone marrow transplantation studies between the NTN-susceptible WKY and NTN-resistant LEW rats together with mesangial cell activation assays show that resident renal cells account for about 10% of glomerular crescent formation (90% of the Crgn susceptibility is caused by circulating cells). The intrinsic renal contribution may be, in part, due to mesangial cell secretion of MCP-1 upon stimulation with TNF-α [4], [7] since WKY cells secrete increased levels of MCP-1 when compared with LEW mesangial cells indicating that there is a genetic predisposition for mesangial cell activation [7].

MCs are modified smooth muscle cells that provide structural support for glomerular capillary loops [12]. They are stromal cells of mesenchymal origin and may therefore have properties in common with bone marrow derived mesenchymal stem cells (MSCs). These latter have been used in cell transfer studies to modify experimental glomerulonephritis in the rat. Elegant studies by Kunter et al. showed that MSC injection resulted in rapid recovery from mesangiolysis in rat mesangioproliferative anti-Thy1.1 glomerulonephritis [13]. The same group used a similar approach in a more progressive model of glomerulonephritis where the Thy1.1 model was aggravated with previous uninephrectomy of the rats [14]. In this model, MSCs prevented progressive renal failure but resulted in the formation of adipose tissue within the glomeruli of the injected rats. Another study showed that human MSCs home to and promote repair of pancreatic islets and renal glomeruli in diabetic NOD/scid mice [15]. Although these studies showed beneficial effect of MSC injection on the pathophysiology of experimental glomerulonephritis, the mechanisms by which MSCs exert their modulatory function are still under investigation and recent reports underlie the importance of cell-to-cell communication through the release of soluble factors by these cells [16].

The secretion of soluble factors together with the inflammatory profile to which MSCs are exposed constitute key aspects of their immunomodulatory activity [17] which could also contribute to renal repair [18]. Notably, monocyte/macrophages have been shown to play an important regulatory role in enabling MSCs to acquire their immunosuppressive activity [19], [20].

Here we have hypothesised that MCs, given their mesenchymal stromal origin, will acquire regulatory properties in the inflammatory milieu generated by infiltaring macrophages at an early stage of Crgn in the WKY NTN. We have also explored the genetic determinants of this mesangial cell regulation by using MCs from NTN-susceptible and NTN-resistant rats. We show the immunosuppressive capacity of rat MCs on splenocyte proliferation. We also demonstrate that the transcriptome of mesangial cells from WKY and NTN-resistant Lewis rat is distinct and this translates into different regulatory effects on macrophages from the same strains; skewing them towards M1 or M2 activation according to their genetic background. We also show that MCs and MSCs differ in their macrophage regulatory properties. We therefore propose that kidney mesangial cells could have unique immunomodulatory effects in “fine tuning” macrophage activation.

## Results

### Rat mesangial cells suppress splenocyte proliferation

**Figure 1:**
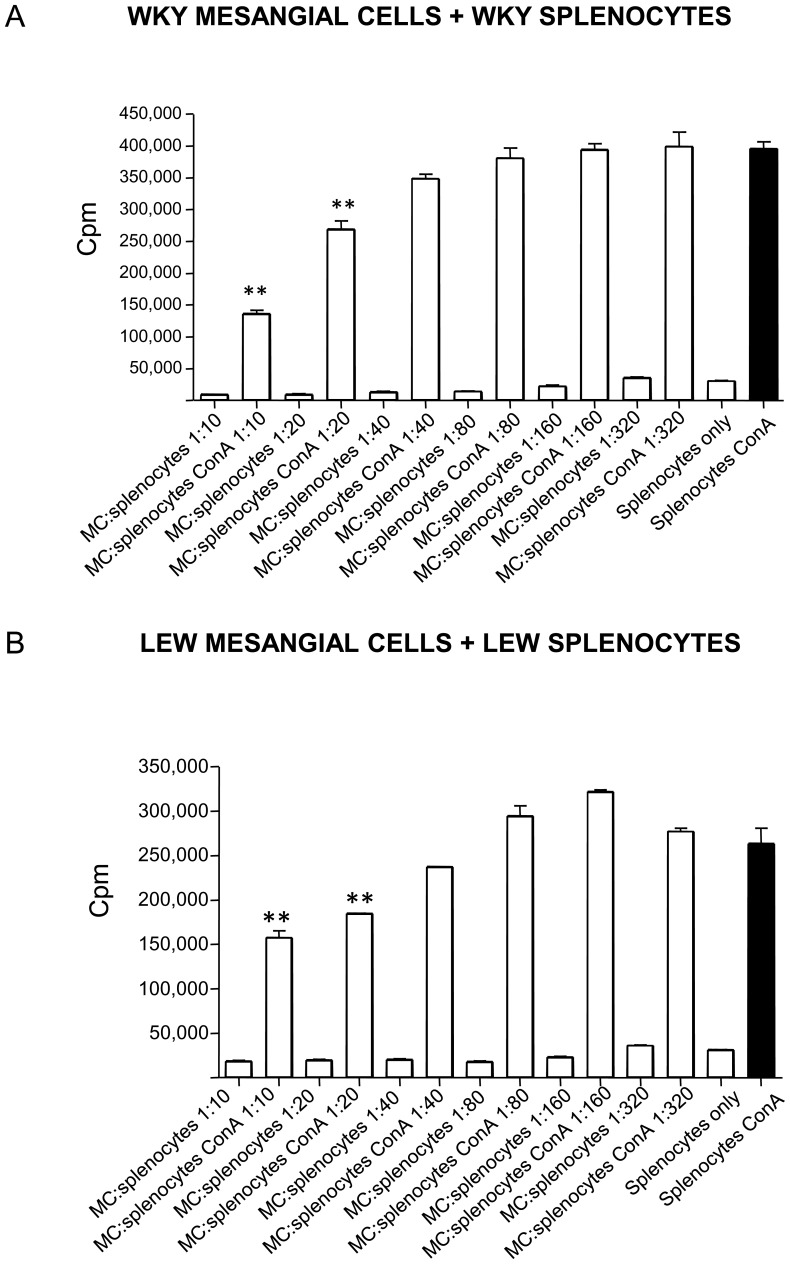
Mesangial cells (MCs) inhibit splenocyte proliferation. Rat MCs were cultured until passage 5 (P5) and incubated with Con-A stimulated or non-stimulated splenocytes for 72 hours. Cell proliferation was measured by incorporation of tritiated thymidine (^3^H-TdR). **A**. Co-culture of WKY MCs and splenocytes resulted in a significant decrease in ConA-stimulated splenocyte proliferation in both 1∶10 and 1∶20 ratio of MC:Splenocyte. **, P<0.001 compared with splenocytes alone. The results are representative of three independent experiments. Cpm, counts per minute **B**. Co-culture of LEW MCs and splenocytes resulted in a significant decrease in Con-A-stimulated splenocyte proliferation in both 1∶10 and 1∶20 ratio of MC:Splenocyte. **, P<0.001 compared to splenocytes alone. The results are representative of three independent experiments. Cpm, counts per minute.

Since the ability of MSCs to suppress splenocyte proliferation was previously described [21], we tested whether mesangial cells can suppress splenocyte proliferation with ^3^H thymidine immunosuppressive assay. We have assessed WKY splenocyte proliferation in presence of WKY mesangial cells and similarly LEW splenocyte proliferation in presence of LEW mesangial cells (Figure 1A and B). Both strains’ mesangial cells suppressed significantly their respective splenocyte proliferation showing that mesangial cells suppress splenocyte proliferation *in* *vitro* (Figure 1A and B).

### Rat mesangial cell transcriptome is genetically determined

**Figure 2:**
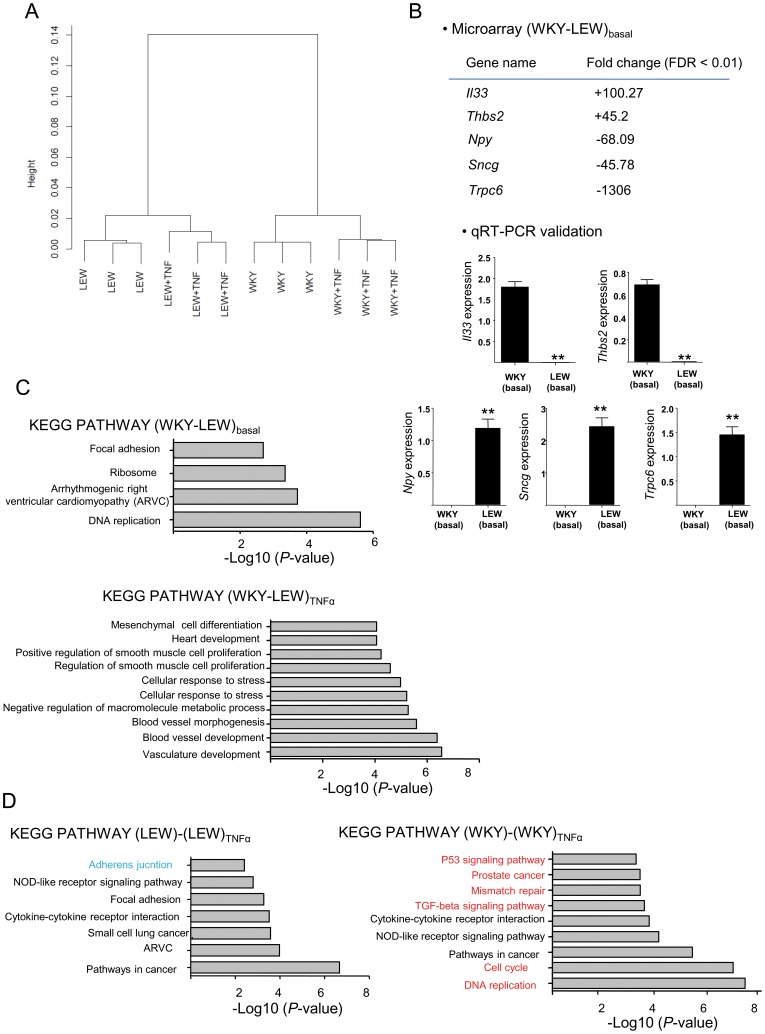
Genome-wide expression analysis by microarrays identifies two distinct transcriptomes in WKY and LEW MCs. **A**. Dendogram representing the hierarchically clustering using all differentially expressed genes between WKY and LEW MCs in basal (unstimulated) and TNFα-stimulated cells. This shows that the strain (WKY or LEW) and the treatment (basal or TNFα-stimulated) effects cluster in 4 distinct groups. **B**. Validation of the markedly differentially expressed transcripts (fold change >10) by qRT-PCR. The upper panel shows the top differentially expressed candidates identified by microarray analysis with a false discovery rate (FDR) <0.01. The positive fold change (FC) values designate up-regulation in WKY MCs and negative FC values designate up-regulation in LEW MCs in basal conditions. The lower panel shows qRT-PCR validation for the transcripts showing differential expression in the microarray dataset. n = 4 rats, **, P<0.001. **C**. KEGG pathway analysis applied to differentially expressed genes between WKY and LEW MCs in basal state (WKY-LEW)_basal_ and (WKY-LEW)_TNFα_ identified the DNA replication and the vasculature development pathways as the most significant ones respectively. **D**. KEGG pathway analysis in LEW and WKY MCs treated with TNFα [(LEW)-(LEW) _TNFα_ and (WKY)-(WKY)_TNF_] identified strain-specific pathways (shown in blue in the LEW and red in WKY) upon TNFα stimulation. Note that the DNA-replication pathway is the most significant one in the WKY MCs following TNFα stimulation.

We have previously shown that NTN-susceptible WKY MCs secrete relatively higher levels of MCP-1 when compared with LEW in both basal and TNFα stimulated MCs, suggesting that there is a genetically determined mesangial cell activation [4], [7]. To study this further, we have performed genome-wide expression analysis by microarrays in control (basal) and TNFα-stimulated mesangial cells derived from WKY and LEW rats. WKY and LEW MC transcriptomes in basal and activated states formed four distinct clusters in the hierarchical clustering analysis (Figure 2A). Although the treatment effect (TNFα) clustered differently from control (basal) samples, the biggest clustering (height of the dendogram) was obtained between the inbred rat strains (Figure 2A). Indeed, there were nearly 4000 differentially expressed genes between WKY and LEW MCs in the basal state (FDR <0.05). The top differentially expressed transcripts (Fc >10; FDR <0.01) were validated by qRT-PCR analysis (Figure 2B). When WKY and LEW mesangial cells’ transcriptomes were compared for differential expression, KEGG analysis showed the most significant enrichment for DNA replication (*P* = 2.41×10^−6^). This suggests that genetic determinants of the MC transcriptome reflect a difference in the proliferative status of WKY and LEW MCs (Figure 2C). Notably, TNFα treatment resulted in a significant differential expression in 1524 genes in WKY MCs but only 804 in LEW MCs, indicating also a genetic difference in response to TNFα stimulation. When LEW and WKY MCs are stimulated with TNFα, there are specific strain-specific KEGG pathways showing significant enrichment in microarray datasets (Figure 2D).

### Mesangial cells shift macrophages to either M1 or M2 activation state depending on the genetic background of the cells

**Figure 3:**
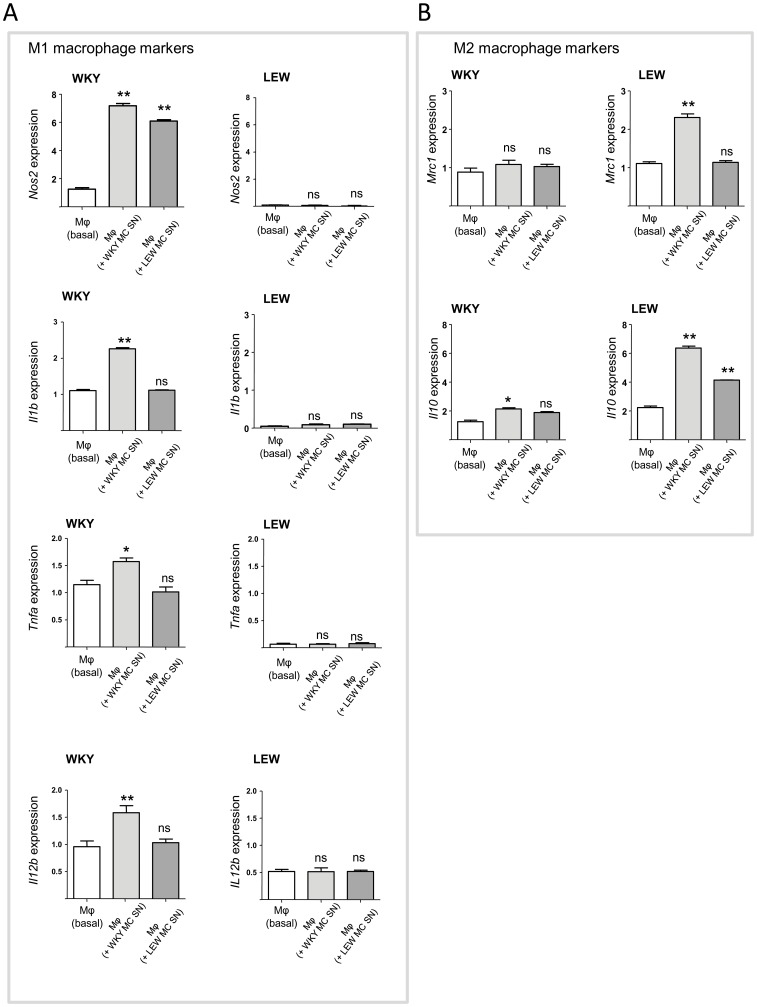
Mesangial cells contain soluble factors polarising macrophages depending on their genetic background. Supernatant transfer from P5 MCs (WKY or LEW) onto WKY or LEW bone marrow-derived macrophages (BMDMs). **A**. qRT-PCR assessment of M1 macrophage markers (*Nos2*, *Il1b*, *Tnfa*, *Il12b*) in WKY (left panel) and LEW (right panel) BMDMs following incubation with MCs supernatants from both strains. Note the increased expression of all M1 markers in WKY BMDMs following incubation with WKY MC supernatants. **B**. qRT-PCR assessment of M2 macrophage markers (*Mrc1*, *Il10*) in WKY (left panel) and LEW (right panel) BMDMs following incubation with MCs supernatants from both strains. Note the increased expression of all M2 markers in LEW BMDMs following incubation with WKY MC supernatants. *, P<0.01; **P<0.001; the results are representative of two experiments, n = 4 rats/strain used per experiment.

In order to determine whether mesangial cells secrete soluble factors that shift macrophage expression towards M1/M2 polarisation, we have performed supernatant transfer experiments from rat mesangial cells onto WKY bone marrow derived macrophages (BMDMs). BMDM expression of M1 and M2 markers were assessed by qRT-PCR following transfer of supernatants from mesangial cells. To determine whether the genetic background of macrophages and mesangial cells influence the potential immunosuppressive properties of mesangial cells, these experiments were performed by using WKY and LEW BMDMs incubated with either WKY or LEW MC supernatant (SN). Interestingly, when MC SN from NTN-susceptible WKY rats were transferred onto WKY BMDMs, this has led to a significant increase in the expression of *Nos2*, *Il1b*, *Tnfa*, *Il12b* (Figure 3A). MC SN from NTN-resistant LEW rats did not result in a significant increase in expression of *Il1b*, *Tnfa* and *Il12b* (Figure 3A). When LEW BMDMs were stimulated with MC supernatants, this has not resulted in a significant increase in the expression of all M1 markers (Figure 3A). We next assessed the expression of M2 macrophage markers such as *Mrc1* and *Il10* and showed that WKY MC SN increase LEW BMDM expression of these markers (Figure 3B). In summary these results suggest that WKY MC SN contain soluble factors that polarise macrophage expression towards either M1 or M2 depending of the genetic background of the macrophages. Similar results were obtained when co-culture experiments were performed instead of transferring the supernatant and when supernatant from TNF-α-stimulated cells were incubated with BMDMs (data not shown).

### The effect of bone marrow derived mesenchymal stromal cells on macrophage gene expression

**Figure 4:**
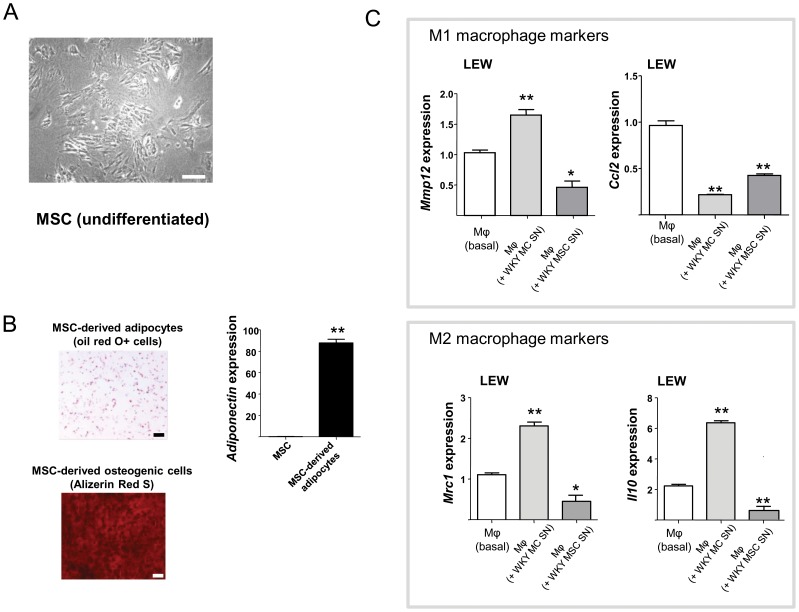
The effect of mesenchymal stem cells on macrophage gene expression. **A**. To characterise rat MSCs, these cells were differentiated into adipocytes and osteogenic cells. The undifferentiated MSCs are shown in light microscopy. Scale bars: 50 µM. **B**. Oil-red-0 and alizarin Red S staining of WKY MSC-derived adipocytes and osteogenic cells (left panel). Adiponectin expression assessed by qRT-PCR (right panel). **P<0.001. Scale bars: 50 µM. **C**. qRT-PCR measurement of M1 (*Mmp12, Ccl2*) and M2 markers (*Mrc1* and *Il10*) in LEW BMDMs incubated with either WKY MC or MSC supernatant. Note the opposite effect of MC and MSC supernatants on *Mmp12*, *Mrc1* and *Il10* expression in LEW BMDMs. **P<0.001, the results are representative of two independent experiments, n = 4 rats/strain used per experiment.

Because of the previously proven immunomodulatory effects of MSCs, and their common stromal origin with kidney mesangial cells, we hypothesised that mesangial cells and MSCs could have similar effects on macrophage gene expression. To test this, we cultured bone marrow-derived MSCs and transferred the supernatants from WKY MSCs to BMDMs to compare the gene expression in BMDMs following incubation with MC supernatant (Figure 4). First we show that rat bone-marrow derived MSC differentiate successfully into adipocytes and osteogenic cells (Figure 4A and B) assessed by presence of oil-red-O+ and Alizarin Red S-stained calcium deposits respectively (Figure 4B). qRT-PCR expression of adiponectin on MSC-derived adipocytes further confirmed the adipocyte differentiation (Figure 4B). MC and MSc SN had contrasting effects on the expression levels of *Mmp12*, *Mrc1* and *Il10* expression (Figure 4C). While MC supernatant from WKY cells selectively increase LEW BMDM *Mrc1* and *Il10* expression (Figure 3B), the same macrophages incubated with WKY MSC supernatant resulted in a significant decrease in the expression of these transcripts (Figure 4C). This suggests that MSCs and MCs have opposite effects on macrophage expression of the selected M2 markers.

## Methods

### Animals

WKY (WKY/NCrl) and LEW (LEW/Crl) rats were purchased from Charles River UK. All procedures were performed in accordance with the United Kingdom Animals (Scientific Procedures) Act, 1986. All the procedures were approved by the Home Office UK.

### Bone marrow-derived macrophage culture

Bone marrow-derived macrophages were obtained and characterised as described previously [4]. Bone marrow-derived cells were allowed to differentiate in Dulbecco's Modified Eagle Medium (DMEM, Gibco) containing 25 mM HEPES buffer (Sigma), 25% L929-conditioned medium, 25% Fetal Bovine Serum (Biosera), Penicillin (100 U/ml; Gibco) and Streptomycin (100 µg/ml; Gibco) and cultured for 5 days on petri dishes (Nunc). Cells were characterised as macrophages by performing immunohistochemistry on methanol-aceton fixed cells for rat ED-1 (Serotec, Oxford, UK) followed by an HRP-labelled anti-mouse polymer development system (Dako Ltd, UK). We found that >99% of the differentiated cells were ED-1+ macrophages and all the primary macrophages used in our study were Cd11b+ (Figure S1).

### ^3^H thymidine immunosuppressive assay

In order to measure the anti-proliferative ability of rat WKY or LEW mesangial cells, these cells were plated at 5×10^4^ cells/well in a 96 well flat bottom plate (Costar), and either 5×10^5^ Con-A stimulated or non-stimulated splenocytes were added for 72 hours. Cell proliferation was measured by incorporation of tritiated thymidine (^3^H-TdR). Cell cultures were pulsed with 0.5 µCi/well of ^3^H-TdR (Amersham, UK) and incubated for the last 18 hours. Cells were then harvested onto filters (Wallac Perkin Elmer, USA) using a 96-well cell harvester. The results of the incorporated thymidine were expressed in counts per minute (cpm) and were a mean of triplicate.

### Kidney mesangial and bone marrow-derived mesenchymal stromal cell culture

Glomeruli from WKY and LEW rats were isolated by sieving as previously described [4]. Purified glomeruli were digested with collagenase type 1 (Sigma) for 20 min. The partially digested glomeruli were cultured in 25-cm^2^ tissue culture flasks in RPMI 1640 medium (Invitrogen) which contained 20% FCS (Biosera), 10% Penicillin/Streptomycin (Invitrogen), L-glutamine (2 nM, Invitrogen) and insulin-transferrin-selenite (Sigma). The cultures were maintained in 37°C, 5% CO_2_ incubators for 6 days. Medium was changed every 2 to 3 days thereafter. By day 21 to 30, cells were subcultured when they reached confluence. These cells were characterized by immunofluorescence staining using cells that were cultured on coverslips. They were positive for Thy-1.1 antigen, myosin, and desmin and negative for pancytokeratin, OX-1, ED-1, and OX-23.

For bone marrow-derived mesencymal stromal cell (MSC) culture, femurs and tibias from WKY and LEW rats were isolated and flushed with Hank’s balance salt solution (HBSS; Gibco). 20×10^6^ bone marrow-derived cells were incubated in one T150 tissue culture flask in MesenCult MSC Basal Medium (Stemcell) that contained MSC stimulatory supplement and 0.5% Penicillin/Streptomycin (Invitrogen). The cultures were maintained in 37°C, 5% CO_2_ incubators their media was changed every 3–6 days. After P5, cells were incubated in StemPro Adipogenesis Differentiation Kit (Invitrogen) and characterized by Oil Red O and 2% Alizarin Red S as MSCs by light microscopy. MSCs were further characterised as Cd11b(−) cells (Figure S1).

### Microarray sample preparation and data analysis

Total RNA was extracted from mesangial cells (P4) using the TRIzol (Invitrogen) method and purified using RNeasy Plus spin columns (Qiagen). 100 ng of total RNA was amplified, labelled and hybridised to Rat Gene 1.0 ST arrays (Affymetrix, Santa Clara, CA, USA) using the Ambion WT Expression Kit (Life Technologies) as per manufacturer’s instructions. Mesangial cells from 3 different biological replicates were used for each strain. For TNFα stimulation, total RNA extracted from mesangial cells incubated over-night with DMEM media containing only recombinant rat TNFα (2 ng/ml) were used. CEL intensity files were produced using GeneChip Operating Software version 1.4 (Affymetrix) and quality tested using the Affymetrix Expression Console v1.1.2. All 12 files were suitable for further analysis. Probe-level data was normalised using robust multichip average (RMA) [22]. A custom definition file was created using up-to-date probe information [23] and filtered to exclude probes containing the 2,520,602 single nucleotide polymorphisms present between the WKY and LEW genomes. The moderated T test with 40,000 permutations implemented in Statistical Analysis of Microarrays (SAM) version 3.0 was used to identify differentially expressed genes at an FDR threshold of 5%. KEGG pathway analysis was carried out using the functional annotation tools within DAVID, the Database for Annotation, Visualisation and Integrated Discovery v6.7 [24]. Ward’s linkage algorithm [25] was used for hierarchical clustering. Dendrograms were calculated with average linkage hierarchical clustering using Spearman rank correlation with Ward metric.

### Quantitative RT-PCR

All qRT-PCRs were performed with an ViiA 7 Real-Time PCR System (Life Technologies). A two-step protocol was used as previously described [26] beginning with cDNA synthesis with iScript select (Bio-Rad) followed by PCR using The Brilliant II SYBR Green QPCR master mix (Agilent). A total of 10 ng of cDNA per sample was used. ViiA 7 software (Life Technologies) was used to determine the Ct values. Results were analysed using the comparative Ct method and each sample was normalised to the reference gene Hprt, to account for any cDNA loading differences. The primer sequences used for the qRT-PCR validation of microarray data are available upon request.

### Supernatant transfer experiments

4 × 10^4^ WKY and LEW mesangial or mesanchymal stem cells were plated in 24-well tissue culture plate (Nunc) in their respective medium and cultures were maintained in 37°C, 5% CO_2_ incubators for 2 days. Both mesangial cells and mesanchymal stem cells were made quiescent in serum-free medium for 24 h and the mesangial cell and mesanchymal stem cell supernatants were then transferred onto bone marrow derived macrophage cultures plated in 6-well tissue culture plate (Nunc) at a density of 1×10^6^ cells/well. The BMDMs were incubated in 37°C, 5% CO_2_ for an additional 24 h. Cell layers were collected for qRT-PCR analysis of M1 and M2 transcripts.

### Statistical analysis

Results are expressed as mean ± SEM. In supernatant transfer experiments, statistical differences in mean values between the unstimulated macrophages (basal) and MSC or MC stimulated cells were compared using a one-way ANOVA with Newman-Keuls Comparison test.

## Discussion

The immunosuppressive properties of MSCs have been widely studied both *in vitro* and in animal models of immune-mediated disorders and clinical trials are currently underway that employ MSCs to treat human immunological diseases [27]. The various molecular mechanisms mediating the immunomodulatory effect of MSCs on different immune cells are still unclear and there are now several reports aiming to elucidate the MSC-macrophage interaction in pathology. ‘MSC-educated’ macrophage activation has been proposed by several groups [28]–[30], and recently, transplantation of mesenchymal stem cells promoted an alternative pathway of macrophage activation and functional recovery after spinal cord injury [31]. Although there is a large body of evidence on the immunomodulatory effects of MSCs, the genetic determinants of the immunosuppressive effects of these cells is largely ignored.

In the kidney, mesangial cells are of stromal origin and share a multitude of phenotypic markers with MSCs such as alpha smooth muscle actin. To date, the role of mesangial cells in macrophage-dependent crescentic glomerulonephritis in general was equally widely studied and there are also reports describing specifically mesangial cell-macrophage interaction *in* *vitro* that occur during the glomerular inflammation [32], [33]. Cytokine secretion by MCs is thought to play a major role in the pathogenesis of glomerulonephritis. Our previous work indicated that mesangial cell activity could be genetically determined as the NTN-susceptible WKY MCs secrete markedly increase amounts of MCP-1 when compared with NTN-resistant LEW rats [7]. These results suggested that mesangial cells could have immunomodulatory properties affecting the infiltration and activation of the macrophages within the inflamed glomerulus. Likewise, we have shown that macrophage function is genetically determined in [WKY × LEW] rats and is a determinant of their susceptibility to macrophage-dependent Crgn [34], [35].

In this manuscript, we asked the following question: Do kidney mesangial cells have similar regulatory effects as mesenchymal stem cells and if so what is the effect of the genetic background on macrophage polarisation?

We show that mesangial cells can suppress splenocyte proliferation as was previously shown for MSCs [36]. We also determined the transcriptome of WKY and LEW MCs showing marked differences in gene expression which suggests that it is under genetic control. Supernatant transfer experiments from mesangial cells onto macrophages shows that soluble factors present in WKY MC supernatant could differentiate macrophages into M1 or M2 depending on the genetic background of these cells. Interestingly the NTN-susceptible WKY BMDMs differentiate into a pro-inflammatory M1 (increase in *Il1b*, *Nos2*, *Tnfa*, *Il12b* expression) phenotype whereas the NTN-resistant LEW BMDMs differentiate into an anti-inflammatory M2 phenotype (increase in *Mrc1* and *Il10*) following incubation with WKY MC supernatant. This suggests that the WKY MC supernatant contain soluble factors that will polarise macrophage activation according to their genetic background. In keeping with this, the genetic background of MCs is important when polarising the macrophages. Indeed, MC supernatants from different genetic backgrounds differentially polarise WKY macrophages towards an M1 phenotype. These results add a novel genetic aspect to the modulatory properties of stromal cells. Our results also suggested that MSCs and MCs have distinct effects on macrophage M1 and M2 polarisation underlying the importance of the possibly unique regulatory effects of MCs in the kidney. MSCs and MCs polarise macrophages in different ways regardless of the genetic background suggesting the existence of epigenetic and/or environmental factors responsible for this effect.

In summary, these results show the importance of genetic determinants of mesangial cell activation in glomerular inflammation. This must be taken into consideration in understanding the unique regulatory effects of these cells on activated macrophages in the pathophysiology of crescentic glomerulonephritis.

## Supporting Information

Figure S1 **Flow cytometry analysis of CD11b surface expression shows that primary rat macrophages (Mϕ, upper panel) and MSCs (lower panel) used in this study are Cd11b+ and Cd11b- respectively.**
(TIF)
